# Nurse prescribing for selected UTIs in Spain: a challenging step forward

**DOI:** 10.1093/jacamr/dlaf102

**Published:** 2025-06-06

**Authors:** Enrique Castro-Sánchez, Aina Huguet-Torres, Aina María Yáñez-Juan, Miquel Bennasar-Veny

**Affiliations:** NIHR Health Protection Research Unit in Healthcare Associated Infections and Antimicrobial Resistance, Imperial College London, London, UK; Research Group on Global Health, University of Balearic Islands, Palma, Spain; Research Group on Global Health, University of Balearic Islands, Palma, Spain; Department of Nursing and Physiotherapy, University of Balearic Islands, Palma, Spain; Research Group on Nursing, Community and Global Health, Health Research Institute of the Balearic Islands (IdISBa), Palma, Spain; Research Group on Global Health, University of Balearic Islands, Palma, Spain; Department of Nursing and Physiotherapy, University of Balearic Islands, Palma, Spain; Research Group on Nursing, Community and Global Health, Health Research Institute of the Balearic Islands (IdISBa), Palma, Spain; Research Group on Global Health, University of Balearic Islands, Palma, Spain; Department of Nursing and Physiotherapy, University of Balearic Islands, Palma, Spain; Research Group on Nursing, Community and Global Health, Health Research Institute of the Balearic Islands (IdISBa), Palma, Spain; CIBER de Epidemiología y Salud Pública (CIBERESP), Carlos III Institute of Health (ISCIII), Madrid, Spain

## Abstract

This paper examines the implications of Spain’s recent legislative reform enabling nurses to prescribe antibiotics for uncomplicated urinary tract infections (UTIs) in women, positioning it as a critical development in antimicrobial stewardship (AMS) and nursing practice. The paper aims to assess how this policy aligns with international models of nurse prescribing, evaluating its potential to enhance workforce flexibility, and identifying some challenges and opportunities regarding its implementation. While this reform may reduce pressures on general practitioners and optimize primary care delivery, it raises questions regarding diagnostic accuracy, restricted formularies and the need for rigorous surveillance. The viewpoint highlights the importance of robust training in diagnostic and clinical reasoning, regular updates to antibiotic formularies and comprehensive auditing mechanisms to ensure safe and effective prescribing. Additionally, we explore broader considerations, such as professional incentives and inter-professional collaboration, essential for the sustained impact of nurse prescribing. By centring the discussion on nursing leadership in AMS, we highlight an underexplored area of policy implementation, with implications for other countries considering similar reforms.

## Introduction

**Figure 1.**
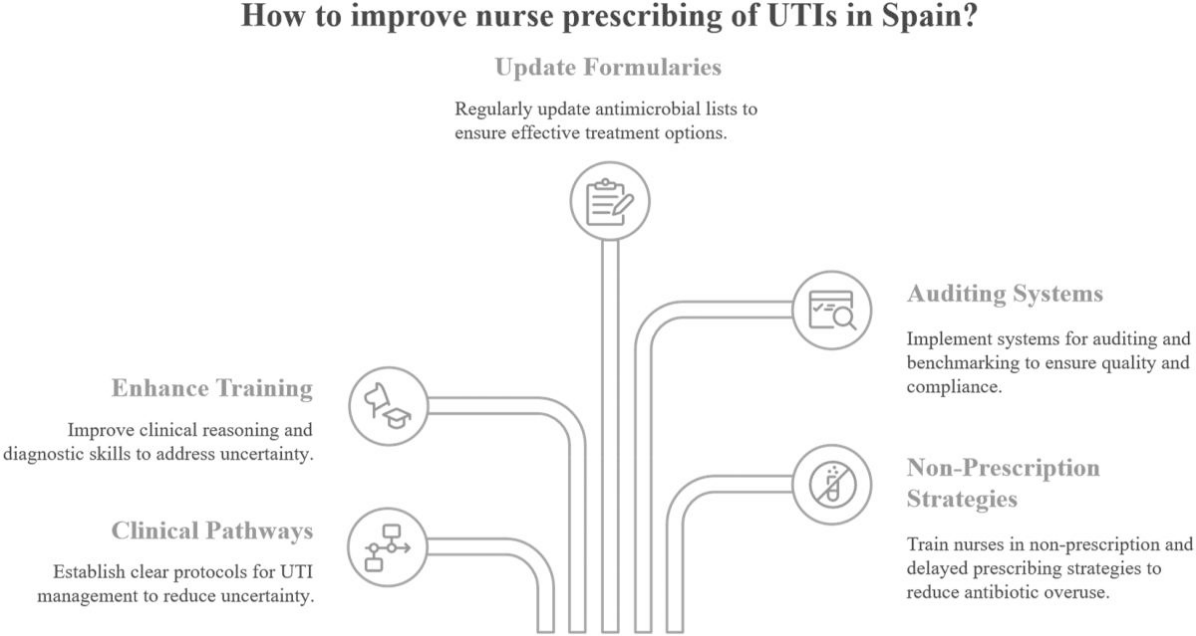
Challenges and recommendations, nurse prescribing of UTIs in Spain.

The recent legislative advancement in Spain, which allows nurses to prescribe certain antibiotics for the management of uncomplicated urinary tract infections (UTIs) in women, represents a significant step forward for the nursing profession in the country.^1^ This paper aims to critically assess the implications of this policy change, examining how it aligns Spain with international models of nurse prescribing, its potential to support antimicrobial stewardship (AMS) initiatives such as the Spanish Plan against Antimicrobial Resistance,^2^ and the challenges associated with its implementation. While this development facilitates timely and effective patient care, concerns persist regarding diagnostic accuracy, AMS, and the broader implications for nursing and healthcare practice in Spain. Addressing these concerns is essential to ensuring the initiative’s success, safety and sustainability (Figure 1).

## A progressive step for nursing in Spain

Empowering nurses to prescribe antibiotics for UTIs is a pivotal moment in the recognition and expansion of nursing competencies, optimizing workforce distribution in response to changing population health needs^3^ and global workforce sustainability debates.^4^ In many European countries, including the UK, the Netherlands and Sweden, nurses have long held prescribing rights within specific clinical frameworks.^5,6^ The shift in Spain modernizes the role of nursing within its healthcare system and aligns it with international best practices, fostering greater autonomy and efficiency in primary care.^7^

The benefits of nurse prescribing are well-documented. Studies suggest that in some contexts, nurse prescribers can achieve comparable or even superior patient outcomes to physicians, including increased adherence to clinical guidelines and patient satisfaction.^8^ The Spanish legislation may reduce consultation times and alleviate the burden on general practitioners, allowing them to focus on more complex cases. UTIs are one of the most common causes of consultation in primary care, and one in 20 Spanish women would their doctor due to urinary symptoms such as frequency and dysuria.

In Spain, it is also not uncommon for patients with uncomplicated UTIs to present at Accident & Emergency (A&E) rather than seeking primary care appointments, as A&E offers relatively immediate access without prior booking.^9^ This practice reflects a broader pattern of healthcare-seeking behaviour that contributes to inefficiencies within the Spanish National Health System.^10^ Currently, despite the non-emergency nature of these cases, nurses in A&E can only play a limited role, primarily triaging patients before referring them to attending physicians. Again, expanding nurse prescribing competencies for UTIs could potentially optimize workforce skill-mix and alleviate pressures on emergency departments.

## Challenges and considerations

### Uncertainty around nurse-led UTI management scope

While this legislative change is progressive, uncertainty remains regarding the extent to which nurses currently manage UTIs in primary care settings. Data on the proportion of women diagnosed and treated for UTIs exclusively by nurses in Spain is limited. Without clear epidemiological data, it is difficult to assess the immediate impact of this initiative on healthcare delivery. Establishing robust surveillance mechanisms and conducting periodic audits will be essential to understand the practical application of this new competency.

### Diagnostic challenges in UTI identification

Accurate diagnosis of UTIs can be complex, particularly given the overlap of symptoms with other conditions such as sexually transmitted infections and interstitial cystitis.^11^ While nurses are well-equipped to identify common symptoms such as dysuria, urgency and frequency, they may lack systematic training in differential diagnosis—an essential competency for safe prescribing. Additionally, although the recently passed legislation includes a general clinical pathway, it remains to be translated into detailed, nurse-appropriate protocols that incorporate structured decision-making algorithms, thereby minimizing diagnostic uncertainty and ensuring safe prescribing practices. Appropriately, the clinical pathway included in the Spanish legislation just passed does not require the use of dipstick urine testing due to its poor diagnostic yield in isolation.^12^ However, such practice is deeply ingrained in nursing practice.^13^ Over-reliance on empirical treatment without confirmatory diagnostics risks both over-prescription and under-diagnosis of more severe infections, a challenge that applies broadly across all clinical professions. Therefore, training in clinical reasoning and diagnostic uncertainty, combined with access to emerging rapid point-of-care tests,^14^ will be crucial to ensure safe and appropriate prescribing.

### The fixed list of antimicrobials: a double-edged sword

The legislation provides a pre-defined list of antibiotics, including fosfomycin and nitrofurantoin, which aligns with European recommendations for first-line treatment of uncomplicated UTIs.^15^ However, antimicrobial resistance patterns are evolving, and a fixed list for nurses may become inadequate over time. Surveillance data indicate rising resistance rates to fosfomycin in certain regions, potentially limiting its efficacy as a first-line treatment in the future.^16^ We have recently seen the approval of gepotidacin tablets for the treatment of female adults with uncomplicated UTIs caused by susceptible microorganisms,^17^ which Spanish nurses would not be allowed to use. A flexible, regularly updated formulary that reflects local resistance patterns is necessary to ensure optimal treatment choices; much better perhaps would be an open formulary as in the UK.

### The need for auditing, benchmarking and clinical supervision

To ensure responsible antimicrobial prescribing by nurses, mechanisms for auditing and benchmarking clinical practice must be established. Monitoring prescribing patterns and adherence to clinical guidelines is vital to AMS.^18^ Countries with established nurse prescribing models have implemented oversight frameworks to assess the safety, effectiveness, and appropriateness of nurse-led antibiotic prescriptions. Perhaps because of such frameworks, the performance of nurse prescribers in primary care regarding antimicrobials is of high quality.^19^ Moreover, national evaluation systems should be implemented from the outset, with specific indicators including prescription adequacy, recurrence rates and patient satisfaction.

### The biggest challenge: deciding not to prescribe

One of the most significant challenges in AMS is the decision *not* to prescribe antibiotics.^20^ Such a challenge may even be more significant for Spanish nurses, despite the clear boundaries that have been set in the recently passed legislation, as patients may at times struggle to understand why their symptoms are managed differently than on previous occasions, or by different health professionals, which may result in frustration. Such frustration could then disincentivize nursing attempts to implement non-prescription strategies, such as delayed prescribing and symptom-based management, which can be effective in many cases of uncomplicated UTIs.^21^ However, there is limited evidence on how well nurses apply these strategies in practice. Additional training in shared decision-making, communication strategies, and patient education will be necessary to equip nurses with the skills to navigate AMS responsibly.

### Beyond clinical considerations: incentivising adoption and ensuring equity

Beyond the clinical challenges, implementation efforts must address the structural and professional conditions that influence the uptake of prescribing roles. These include workload pressures, legal liability concerns and insufficient remuneration or recognition. Ensuring appropriate incentives—financial, professional and educational—will be key to successful integration of these competencies. Moreover, this policy change also bears gender and equity implications: by addressing a condition predominantly affecting women and often trivialized in healthcare, nurse prescribing may contribute to more equitable and responsive care models.

## Future directions

To ensure long-term sustainability, future evaluations should explore the longitudinal impact of nurse prescribing on resistance trends using sentinel surveillance data and regional microbiological reporting systems. In parallel, qualitative studies on nurses’ experiences with diagnostic uncertainty and prescribing responsibility could deepen our understanding of barriers and enablers to effective practice. Research should also focus on the impact of nurse-led prescribing on inter-professional collaboration, especially in primary care teams, and how prescribing rights reshape professional identities and hierarchies.

## Conclusion

Spain’s decision to authorize nurse prescribing for uncomplicated UTIs in certain women is a progressive step that aligns with international practices and strengthens the nursing profession. However, its success hinges on addressing key clinical challenges, including diagnostic accuracy, antimicrobial resistance and prescribing oversight. Establishing robust auditing mechanisms, swiftly updating legislation to incorporate formularies based on local resistance patterns, and equipping nurses with training in non-prescription strategies will be essential to optimizing patient care while maintaining responsible antimicrobial use.

Nurses have traditionally been absent from AMS interventions, yet there is a growing demand for their participation and leadership in this area.^22^ The approval of these prescribing rights strengthens their contribution and requires that national AMS plans do not consider this group as an afterthought. To fulfil the promise of this reform, implementation strategies must be inclusive, well-resourced, and centred on professional development, patient communication, and system-level evaluation.
